# Face Processing in Developmental Prosopagnosia: Altered Neural Representations in the Fusiform Face Area

**DOI:** 10.3389/fnbeh.2021.744466

**Published:** 2021-11-18

**Authors:** Alexa Haeger, Christophe Pouzat, Volker Luecken, Karim N’Diaye, Christian Elger, Ingo Kennerknecht, Nikolai Axmacher, Vera Dinkelacker

**Affiliations:** ^1^JARA-BRAIN, Jülich, Germany; ^2^Forschungszentrum Jülich GmbH, Institute of Neuroscience and Medicine (INM-11), Jülich, Germany; ^3^Department of Neurology, RWTH Aachen University, Aachen, Germany; ^4^Paris Descartes, MAP5, Paris, France; ^5^RWTH Aachen University, Aachen, Germany; ^6^Institut du Cerveau et de la Moelle épinière, ICM, Inserm U 1127, CNRS UMR 7225, Sorbonne Université, Paris, France; ^7^Department of Neurology, Beta Klinik, Bonn, Germany; ^8^Institute of Human Genetics, Westfaelische Wilhelms-Universitaet Muenster, Muenster, Germany; ^9^Department of Neuropsychology, Ruhr University Bochum, Bochum, Germany; ^10^Neurology Department, Hautepierre Hospital, University of Strasbourg, Strasbourg, France; ^11^Rothschild Foundation, Neurology Department, Paris, France

**Keywords:** prosopagnosia, fusiform face area (FFA), perception, representational similarity analysis (RSA), Donsker’s random walk analysis, working memory, face recognition

## Abstract

**Rationale:** Face expertise is a pivotal social skill. Developmental prosopagnosia (DP), i.e., the inability to recognize faces without a history of brain damage, affects about 2% of the general population, and is a renowned model system of the face-processing network. Within this network, the right Fusiform Face Area (FFA), is particularly involved in face identity processing and may therefore be a key element in DP. Neural representations within the FFA have been examined with Representational Similarity Analysis (RSA), a data-analytical framework in which multi-unit measures of brain activity are assessed with correlation analysis.

**Objectives:** Our study intended to scrutinize modifications of FFA-activation during face encoding and maintenance based on RSA.

**Methods:** Thirteen participants with DP (23–70 years) and 12 healthy control subjects (19–62 years) participated in a functional MRI study, including morphological MRI, a functional FFA-localizer and a modified Sternberg paradigm probing face memory encoding and maintenance. Memory maintenance of one, two, or four faces represented low, medium, and high memory load. We examined conventional activation differences in response to working memory load and applied RSA to compute individual correlation-matrices on the voxel level. Group correlation-matrices were compared via Donsker’s random walk analysis.

**Results:** On the functional level, increased memory load entailed both a higher absolute FFA-activation level and a higher degree of correlation between activated voxels. Both aspects were deficient in DP. Interestingly, control participants showed a homogeneous degree of correlation for successful trials during the experiment. In DP-participants, correlation levels between FFA-voxels were significantly lower and were less sustained during the experiment. In behavioral terms, DP-participants performed poorer and had longer reaction times in relation to DP-severity. Furthermore, correlation levels were negatively correlated with reaction times for the most demanding high load condition.

**Conclusion:** We suggest that participants with DP fail to generate robust and maintained neural representations in the FFA during face encoding and maintenance, in line with poorer task performance and prolonged reaction times. In DP, alterations of neural coding in the FFA might therefore explain curtailing in working memory and contribute to impaired long-term memory and mental imagery.

## Introduction

Face recognition is a key skill for social interaction. In developmental prosopagnosia (DP), face recognition is impaired without a history of brain damage and affects about 2% of the population (Kennerknecht et al., 2006, 2008; Bowles et al., 2009), with strong indications for heritability for the ability to recognize faces (McConachie, 1976; Duchaine et al., 2007; Kennerknecht et al., 2008, 2011).

Previous studies have shown that face-processing depends on a complex network of brain modules. A highly influential model by Haxby et al. (2000) divided this network into a core and an extended system. In this model, the core system is constituted of regions within the ventral temporo-occipital “what stream” of object recognition: the inferior occipital gyrus, the superior temporal sulcus and the fusiform face area (FFA) in the lateral fusiform gyrus (Kanwisher et al., 1997; Haxby et al., 2002; Gobbini and Haxby, 2006; Jonas et al., 2015). The extended network encompasses the anterior temporal lobe, as well as limbic and parietal structures (Haxby et al., 2002; Simmons et al., 2010; Nestor et al., 2011; Borghesani et al., 2019). Duchaine and Yovel (2015) revisited and expanded the model in functional and anatomical terms. They delineated the dorsal processing route parting from early visual areas via the posterior and anterior superior temporal sulcus to the inferior frontal gyrus. This route has a particular role in the processing of changeable aspects of face stimuli, such as face motion and gaze direction. The ventral route comprises the occipital face area, the posterior and anterior portion of the FFA and the anterior temporal lobe, with differential role in view, identity and semantic face analysis. Importantly, this model revises the hierarchy and feed-forward concept of the Haxby model and insists on a distributed network interaction. While the occipital face area has indeed temporal precedence over the FFA (Sadeh et al., 2010; Babo-Rebelo et al., 2021), occipital lesions do not disrupt FFA activation and relations are reciprocal (Duchaine and Yovel, 2015). Both models concur in the highly complex architecture and interactions within the face processing network.

Within the framework of this network, it is probably not one deficient module but the alteration of the interaction between many modules that leads to the full clinical picture of DP (Dinkelacker et al., 2011; Zhao et al., 2018). Furthermore, DP does not seem to be a homogeneous monogenetic disease. Rather, it has to be regarded as face recognition abilities below standard performance (Bowles et al., 2009; Barton and Corrow, 2016), which are at least partly running in families (Duchaine et al., 2007; Kennerknecht, 2008; Lee et al., 2010; Bate and Tree, 2017). Nevertheless, we consider that modules specifically implicated in face identity processing merit special interest in DP, and in turn, that DP may unravel neural processing aspects which are indispensable for face expertise.

Our choice to focus on the right FFA was thus motivated by both theoretical and methodological considerations. In theoretical terms, it is widely acknowledged that curtailing in face identity processing is a paramount aspect of DP (Fisher et al., 2017). Face identity is mostly conveyed by the ventral route described above with a special emphasis on the FFA and the anterior temporal lobe (Axelrod and Yovel, 2015; Duchaine and Yovel, 2015; Nordt et al., 2018). Among these structures, the lateral section of the right middle fusiform gyrus shows by far the largest face-specific responses in intracerebral recordings (Jonas et al., 2016). In methodological terms, the right FFA is most robustly activated by face stimuli and hence accessible to in-depth functional analysis (Weiner et al., 2012; Bukowski et al., 2013; Rossion, 2014; Duchaine and Yovel, 2015; McGugin et al., 2018). The anterior temporal face area, on the contrary, is subject to major susceptibility artifacts in fMRI and thus difficult to analyze in a reproducible manner (Jonas et al., 2016).

In the context of working memory for faces, the FFA is strongly implicated, as its activation can be promoted by raising the working memory load (Jha and McCarthy, 2000; Druzgal and D’Esposito, 2001, 2003; Ranganath et al., 2004a, b; Haeger et al., 2015). Combined data pointed to the fact that increased activation goes along with better working memory performance (Lepsien and Nobre, 2007), which obviously is sustained both by the FFA and its interaction with other modules of the face processing network (Lin et al., 2019).

With regard to conventional fMRI activation analysis, some previous studies propose that the general FFA function is preserved in DP (Avidan and Behrmann, 2009; Thomas et al., 2009; Eimer et al., 2012; Avidan et al., 2014; Rosenthal et al., 2017). Conversely, another line of evidence points to reduced and altered blood-oxygenation-level dependent (BOLD) responses in the FFA (Williams et al., 2007; von Kriegstein et al., 2008; Dinkelacker et al., 2011; Furl et al., 2011; Németh et al., 2014; Rivolta et al., 2014; Witthoft et al., 2016; Jiahui et al., 2018).

To resolve these discrepancies, recent advances in computational neuroscience have permitted to go beyond the mere activation level and unravel the fine-tuned neural signature within the FFA. Zhang et al. (2015) applied multivariate pattern analysis to the BOLD signal during face perception. Even when face selectivity was present in the right FFA both in prosopagnosia as well as in controls, multivariate pattern analysis revealed impaired face configural decoding in DP, indicating the central role of the FFA in face expertise, also shown in other studies (Schiltz and Rossion, 2006; Liu et al., 2010; Zhang et al., 2012). Investigation of the neural activity of the FFA during face encoding and maintenance may help to discern fundamental mechanisms of prosopagnosia and explain the etiology of their impaired face memory.

In this study, we therefore use a modified Sternberg paradigm (Sternberg, 1975) to analyze the role of FFA during face processing in DP. So far, advances in neuroimaging have allowed for the detection of changes in brain structure and interregional connectivity of the processing network in DP (Albonico and Barton, 2019). On the functional level, however, detailed analyses within specific face modules are scarce. We therefore go beyond conventional activation level, and apply Representational Similarity Analysis (RSA) (Kriegeskorte et al., 2008), a computational approach that allows to determine correlations of neural responses within brain modules (Lee and Geng, 2017) and therefore to scrutinize neuronal representations of faces on neuronal level. We were further interested, if potential functional impairment was reflected on structural level, by additionally performing volumetric comparisons.

We hypothesized that the capacity to produce and to maintain high precision processing of faces would distinguish subjects with DP (DPs) from typically performing control subjects (CTL) and help to explain deficiencies in face processing in DP which might finally lead to decreased face expertise.

## Materials and Methods

### Subjects

A total number of 25 subjects was included in the present study. The group consisted of 13 (9 female) DPs, mean age 53.2 years (SD ± 12.5; range 23–70 years) and 12 (5 female) CTLs, mean age of 47.6 (SD ± 12.0; range 19–62 years). The age difference was not significant [*t*(23) = 1.15, *p* = 0.26]. Results of the Chi-Square test indicated no significant between-group differences for gender (*p* = 0.17). All subjects were German native speakers.

Our concept of prosopagnosia comprised a functional phenotype, with the definition of a decreased ability to recognize faces, without a history of possibly acquired impairment due to e.g., cerebral lesions or psychiatric impairment. Diagnosis of developmental prosopagnosia was therefore established according to the criteria previously described by Grüter et al. (2008) and based on a multistage procedure comprising three main assessment steps, as also applied by several previous studies for diagnostic purposes (Kennerknecht et al., 2008, 2021; Stollhoff et al., 2010, 2011; Dinkelacker et al., 2011; Esins et al., 2015, 2016; Zhao et al., 2016).

First, all participants filled in a screening questionnaire established by Kennerknecht et al. (2006) to assess prosopagnosia symptoms by 21 items, with items rated on a five-point Likert scale. These 21 items covered one or more of the following nine dimensions: (1) Face recognition, (2) learning/memorizing individual faces, (3) false positive and negative rates of face recognition, (4) general facial information (such as gender, physical attractiveness, and emotions), (5) demonstrating the presence/absence of inner images of familiar faces and/or objects, (6) complex pattern recognition (7) compensatory strategies, (8) socialization, and (9) heritability. For more details on the single sub-items, please see the supplementary of this manuscript as well as (Dinkelacker et al., 2011; Grüter et al., 2011; Johnen et al., 2014; Zhang et al., 2015; Kennerknecht, 2021). Responses that indicated prosopagnosia obtained a higher score of up to 5. A score lying one standard deviation (SD) above the mean score of all subjects was taken as indicative for prosopagnosia (Kennerknecht, 2021). After meeting the screening criteria for prosopagnosia, subjects underwent a standardized interview by an expert (IK) taking 60–90 min. This detailed interview is crucial to exclude other causes for degraded face recognition such as poor eyesight, poor visual acuity, or earlier brain damage (head injury, encephalitis/meningitis, cerebral anoxia/hypoxia, asphyxia, cerebral malformation). The interview assessed anamnestic difficulties in the judgment of gender, attractiveness, or emotional information of faces. Furthermore, individual and family history of psychiatric diseases were inquired, which could be accompanied by agnosias, e.g., Asperger’s syndrome and autistic spectrum disorders. In addition, we asked for other associated cognitive and behavioral deficits, such as sense of orientation, hints to object agnosias, differentiation of inter- and intra-class objects e.g., very well-known plants/tree species or animals/birds species, color blindness, social skills, e.g., number of friends and eye contact (Kennerknecht, 2021). Finally, the manifestation of prosopagnosia was considered to lie along four dimensions, relying on four main clinical symptoms of prosopagnosia as described before (Kennerknecht et al., 2006, 2008, 2021; Grüter et al., 2007; Stollhoff et al., 2010, 2011; Johnen et al., 2014): (1) Uncertainty in face recognition, (2) Significantly prolonged recognition time for faces, (3) Development of compensatory strategies as sign of a longstanding problem, and the (4) Repeated anecdotal stories of events such as having overlooked familiar faces (see also the Supplementary Material for more details on this part of the interview). All subjects additionally conducted as a third final diagnostic test for confirmative purposes the Cambridge Face Memory Test (CFMT), which is commonly used in DP (Duchaine and Nakayama, 2006; Duchaine et al., 2007; Avidan et al., 2011; DeGutis et al., 2014).

Diagnosis of prosopagnosia was therefore based on a complex pattern of features, representing both clinical complaints of long-term memory deficits and compensatory strategies (interview), tests of short-term memory (CFMT), as well as family history for some of the DP-participants. The CFMT revealed a significant difference in accuracy between the prosopagnosic and the control group [*t*(23) = 4.94; *p* = 5.40⋅10^–5^, *d* = 1.99]. Control participants had an average accuracy of 75.90% (SD ± 9.6%, range 59.7–90.0%). Prosopagnosic subjects had an average accuracy of 54.1% (SD ± 12.2%, range 31.9–73.6%) in the CFMT. There was no significant correlation between age and CFMT performance (*r_*s*_* = −0.22, *p* = 0.29). One control participant with a dubious CFMT under chance level (47%) was excluded from the study. One control subject and four DP-participants came from the same family. Two other DP participants had a family history of DP. Other control subjects were chosen among regular participants of the Life and Brain (Bonn, Germany) study control cohort. The study was performed according to the WMA Declaration of Helsinki and approved by the local ethics committee (Protocol No. *3XKenn2* and *DI 1217/2-1*). All subjects gave written informed consent for participation in our study. Control participants were paid for their contribution, DPs received travel funding.

### Experimental Design

The experiment consisted of a modified Sternberg paradigm, using a database of 153 female and 153 male grayscale photos of faces, which had been rated as neutral and tested in fMRI and intracranial recordings of working memory as described in Axmacher et al. (2007). Face stimuli were homogeneous in contrast and congruent with the face ethnicity of the participants (Esins et al., 2015).

These stimuli were presented in three different conditions:

(i) In the low load condition, subjects were presented with one face (either male or female) and three scrambled pictures.
(ii) In the medium load condition, two faces and two scrambled pictures were shown.
(iii) In the high load condition, four faces were presented.

Each trial started with the presentation of a picture (face or scrambled) for 800 ms with an interval of 1,000 ms before the next picture was shown, leading to a duration of 6,200 ms for the picture presentations (4 × 800 ms + 3 × 1,000 ms). Pictures were shown in random order.

**FIGURE 1:**
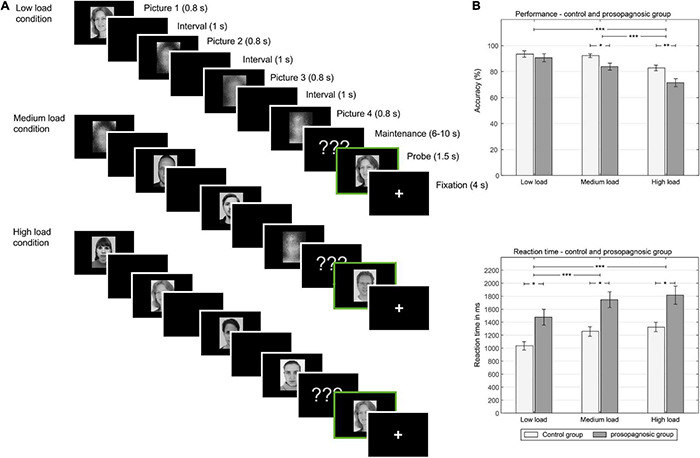
**(A)** Left: One trial of different experimental conditions (low load, medium load, high load memory condition): In the low load condition, one face, and three scrambled pictures are shown in consequent manner, in the medium condition, two faces and two scrambled pictures are shown. In the high load condition, four faces are shown. Between picture presentation a black screen is shown. Male and female faces are not mixed in one trial. After the encoding phase, a maintenance phase is presented. After the maintenance phase with various time durations, a memory probe is shown (indicated here with a green frame). Subjects decide at this point, if the picture is new or old. **(B)** Right: behavioral results: Above, performance in the different conditions low, medium and high load for the control and prosopagnosic group; Below, reaction time in ms for the different conditions. *(*p* < 0.5), **(*p* < 0.01), ***(*p* < 0.001).

After a maintenance phase with a mean duration of 8,000 ms (6,000–10,000 ms), a probe picture was shown for 1,500 ms and subjects had to decide whether the presented face was a new face or an old face, i.e., previously presented in this trial. Before the start of the next trial, a fixation cross was presented for 4,000 ms, leading to a total trial duration of 17,700–21,700 ms. Subjects could answer during the presentation of the probe and during the fixation time before start of the next trial. In total, there were 84 trials (28 for each condition) and 6 randomized breaks of 30 s during the experiment. The total duration of the paradigm was about 30 min. Different faces were used on every trial to prevent long term memory effects, therefore no face in the encoding phase was shown twice. Subjects were asked to respond as fast and accurately as possible and did not receive feedback on their performance during the experiment. Proportion of match/non-match trials was 50:50; thus, a total of 238 (196 for presentation/42 for new probes) unique unscrambled faces were selected out of the 306 available. An overview of the different conditions is shown in Figure 1A (Axmacher et al., 2007). The paradigm was programmed with E-prime (Psychology Software Tools, Pittsburgh, PA) and presented to the subjects via video goggles (NordicNeuroLab, Bergen, Norway) in the scanner. Video goggles were adapted for visual acuity.

### Functional Localizer

We used an independent functional localizer to delineate the FFA in each subject (Kanwisher et al., 1997; Maguire et al., 2001). The localizer used blocks consisting of neutral and positive faces as well as objects and houses, passively viewed by the subjects. Each block consisted of 19 images and had a duration of 19 s. Each of the four categories was shown four times. By forming contrasts between faces and non-face stimuli, FFA activity was individually established within the mask (*wfu_pickatlas*)^1^ of the fusiform gyrus (see below). Spheres of 80 voxels size were drawn around peak voxel activations to create individual masks of equalized sizes, and were used in the consequent analyses of the Sternberg paradigm. An overview of the peak coordinates for each subject is given in Supplementary Table 1, as well as the t-maps of the fusiform activation clusters in our GitHub depository (see below).

### Functional Magnetic Resonance Imaging Data Acquisition

The study was conducted on a 3 T Siemens TRIO MRI scanner (Siemens Medical, Erlangen, Germany). Both T1 structural volume images (TR/TE/TI, 1,570/2,75/800 ms; 160 slices; matrix 256 × 256 mm^2^, spatial resolution 1 × 1 × 1 mmł voxels) as well as T2^∗^-weighted axial echo-planar images with BOLD contrast [gradient echo; TR/TE, 2,700/33 ms; 36 axial slices parallel to anterior commissure-posterior commissure (ACPC) plane; acquired in ascending direction; matrix 64 × 64 mm^2^, field of view 230 mm, slice thickness 2 mm; inter-slice gap 0.5 mm; spatial resolution 1.8 × 1.8 × 2 mmł voxels] were acquired. The experiment contained two functional sessions, the functional localizer followed by the main experiment.

### Statistical Analyses

#### Behavioral Analysis

Response accuracy (“old/new,” percentage of correctness) and reaction times were recorded with E-Prime. Behavioral data were analyzed by first performing a mixed analysis of variance (ANOVA) including subject group (controls vs. prosopagnosics) as well as memory condition (low, medium, high memory load) followed by Student’s *t*-tests. Results were corrected for multiple comparisons via Holm-Bonferroni correction and reported as significant when *p* < 0.05. The Greenhouse-Geisser adjustment was used for correction in case of violations of sphericity. Effect size was stated via Cohen’s *d*. Behavioral analyses were performed via MATLAB and SPSS.

#### Functional Magnetic Resonance Imaging Univariate Analysis

Functional data were analyzed with the Statistical Parametric Mapping toolbox (SPM8; The Wellcome Center for Neuroimaging, London, United Kingdom).^2^ Scans from each participant were realigned using the first scan as a reference. All EPI images were unwrapped, slice time corrected, spatially normalized into MNI standard space using parameters from the segmentation of the T1 structural image (Ashburner and Friston, 2005), resampled to 2 × 2 × 2 mm^3^ voxels and spatially smoothed with a Gaussian kernel of 6 mm FWHM.

### Overview of the MR Analyses Flow

**FIGURE 2:**
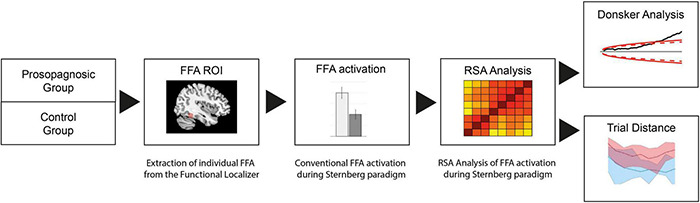
Flow chart of fMRI analysis process: The FFA region is derived from a functional localizer and conventional FFA activation during the Sternberg paradigm extracted. For analysis on voxel-wise level, RSA with a Donsker’s random walk analysis is performed.

Data were analyzed in subsequent steps as detailed in the flowchart of Figure 2 and as described below. We first determined the FFA region from the functional localizer for each individual subject. The right FFA-mask was then applied for the BOLD-images of the Sternberg main paradigm, and (1) FFA activation conventionally extracted for estimation of global activation of this region in both groups for all memory conditions and then (2) an RSA analysis performed to examine the microscale functional architecture of the FFA during the paradigm. The RSA-matrices were then probed for the level of maintenance of neural representations over time by determining the level of correlations as a function of the trial distances. Additionally, functional activation was further linked to behavioral performance of all the subjects.

#### Main Experiment—Sternberg Paradigm

The main experiment, i.e., the Sternberg paradigm, was modeled in an event-related design after convolving each event-related unit impulse (indexing trial onset) with a canonical hemodynamic response function. The encoding phase (onset of stimulus presentation until onset of a maintenance phase) was modeled as an event separate from the maintenance phase (equivalent to 6,000–10,000 ms). In addition to the 6 main regressors for the three load conditions (low, medium, high load) in the encoding and maintenance period, we included a regressor modeling the probe phase and a regressor for all the incorrect trials (Axmacher et al., 2007). One regressor was further created for modeling the breaks. Six movement regressors to account for residual motion artifacts and one linear drift were included in our design matrix. For each subject, condition-specific effects were estimated according to the general linear model. An exemplary design matrix from SPM for one subject is presented in Supplementary Figure 2.

#### Functional Localizer

The Functional Localizer blocked-design modeled each category (neutral faces, positive faces, objects, and houses) as a separate regressor (including time derivatives). Movement parameters and a linear drift regressor were included as additional nuisance covariates. An exemplary design matrix from SPM for one subject is presented in Supplementary Figure 1. Individual FFA activation was defined by contrasting all faces (positive and neutral) vs. non-face stimuli, using a liberal threshold of *p* < 0.001. The activated FFA cluster was then masked by a dilated-by-2-voxels anatomical mask of the fusiform gyrus from the toolbox wfu_pickatlas for SPM (version 3.0.4) to obtain FFA regions individually for each subject (Maldjian et al., 2003; Haeger et al., 2015). Peak activations in this region were used as center for a sphere of 5 mm radius equivalent to 80 voxels in order to obtain masks equal in size for each individual subject. Since subjects showed more pronounced activation in the right FFA than in the left FFA, our focus was on the right hemispheric FFA for all further analyses, also in line with literature showing the central role of the right FFA in face processing (Hoffman and Haxby, 2000; Schiltz and Rossion, 2006; Parvizi et al., 2012; Zhang et al., 2015; McGugin et al., 2018). For three subjects (one control, two prosopagnosic subjects), *p*-value thresholds were lowered to *p* < 0.01 to visualize clusters in right FFA region. In order to exclude that functional differences resulted from differences in size of FFA activation clusters, mean original cluster sizes inside the fusiform gyrus between controls (448 mm^3^; SD 444) and prosopagnosics (249 mm^3^; SD 179) were compared and were not significantly different [*t*(23) = 1.50; *p* = 0.15; *d* = 0.60]. An overview of the peak coordinates of the FFA-regions of each subject is given in our supplementary as well as in our GitHub repository (see below).

#### Representational Similarity Analysis—Step by Step

To examine neural representations reflected by each voxel’s response across trials, we performed a modified representational similarity analysis (RSA; Kriegeskorte et al., 2008). For this analysis, four main steps were performed:

(1) Creation of a new design matrix, defining each trial as single event, to derive voxel activation as a proxy of neural representations.
(2) Extraction of voxel-wise beta values inside right FFA in every subject for every correct trial and creation of a correlation matrix, correlating single-trial beta values within the FFA with the beta values from every other trial.
(3) Donsker’s random walk analysis for comparison of correlation matrices between groups and memory conditions.
(4) Analysis of RSA matrices over time.
(5) Association of RSA results with subjects’ behavioral performance.

#### Step 1 and 2: Creation of Representational Similarity Analysis Design Matrix

As first step, a new design matrix was created according to the design matrix from the first level analysis in which each trial was modeled separately as one regressor. This results in a total of 84 regressors for the maintenance trials, one regressor summarizing all the encoding phases, one regressor for all probes and one regressor for the breaks. This procedure was performed for the analysis of the encoding phase accordingly. For mean correlation matrices and all further analyses, only trials with correct behavioral response were analyzed. Beta images of these trials were then used for extracting single-voxel activation clusters from the individual FFA masks based on the functional localizer. For each subject, we then correlated the single-trial beta values within the FFA with the beta values from every other trial. Considering the data of a single memory load condition (low, medium, high load), the elements $\gamma_{i,j}^{P}$ of the per-subject RSA matrices represent the Spearman’s rank correlation coefficient of subject P between the subject’s correctly answered trials *i* and *j* over all voxels of the subject’s specific FFA mask. Note that for the different memory load conditions, the size of the RSA matrices may differ, as their size is determined by the lowest number of correctly answered trials *T*: 0≤*i*,*j* < *T*, with $T=\min_{\forall p}T^{P}$ and *T^P^* being the number of correctly answered trials of subject P.

**FIGURE 3:**
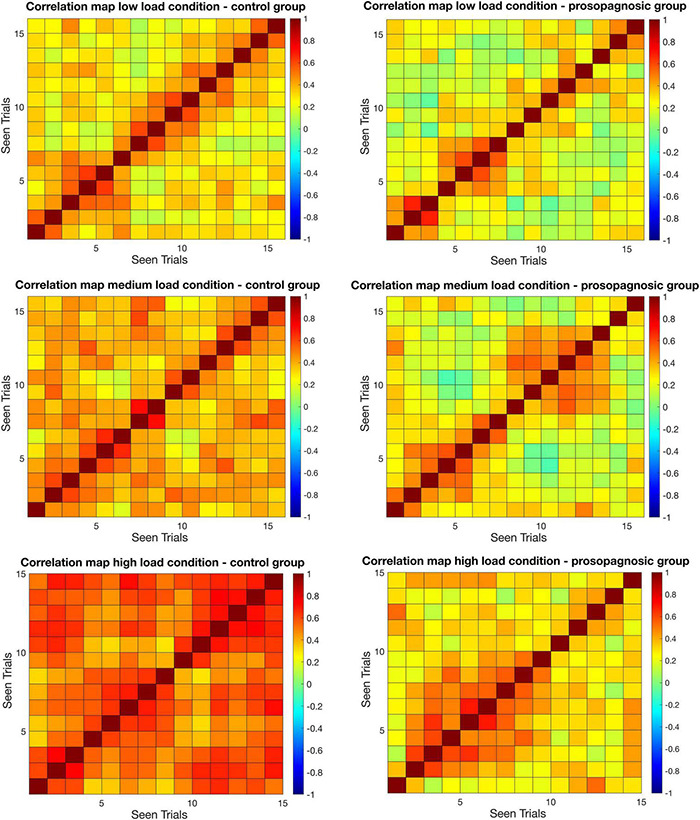
Mean correlation matrices for control and prosopagnosic group for different memory conditions for the maintenance phase. On the *x* and *y* axes, correctly answered trials in chronological sequence ordered by seen are represented. Seen is defined by the chronological order by which trials are presented to the subjects.


RSA was performed separately for the different memory load conditions, resulting in separate correlation matrices: in the low load and medium load condition, matrices consisted of *T* = 16 trials and in the high load condition in *T* = 15 trials, according to the number of correct trials obtained in all subjects. Note that correlation matrices thus represent the correlation of activation in FFA voxels in a sequential series of correct responses and do not necessarily refer to exactly the same trial for individual subjects. For each condition, we determined the group correlation coefficient statistics collapsed over all trials, separately for both groups, healthy controls: *P*^*CTL*^ and prosopagnosic subjects: *P*^*PRO*^ with respective cardinalities *n*^*CTL*^ = |*P*^*CTL*^| and *n*^*PRO*^ = |*P*^*PRO*^|, as mean and standard deviation (Figure 3):

$${\hat{\gamma }}_{i,j}^{CTL}=\frac{1}{n^{CTL}}\sum_{P\in P^{CTL}}\gamma_{i,j}^{P},{\hat{\gamma }}_{i,j}^{PRO}=\frac{1}{n^{PRO}}\sum_{P\in P^{PRO}}\gamma_{i,j}^{P}$$

$${\hat{\sigma }}_{i,j}^{CTL}=\sqrt{\frac{1}{n^{CTL}-1}\sum_{P\in P^{CTL}}{\left(\gamma_{i,j}^{P}-{\hat{\gamma }}_{i,j}^{CTL}\right)}^{2}}$$

$${\hat{\sigma }}_{i,j}^{PRO}=\sqrt{\frac{1}{n^{PRO}-1}\sum_{P\in P^{PRO}}{\left(\gamma_{i,j}^{P}-{\hat{\gamma }}_{i,j}^{PRO}\right)}^{2}}$$

#### Step 3: Comparison of Group Matrices Based on Random Walk Construction and Donsker’s Theorem

Donsker’s theorem (Donsker, 1951), also known as the invariance principle (Pinsky and Karlin, 2011), was applied for the comparison of the group matrices. Comparing matrices amounts to the fact that the elements of the difference matrix are compatible with independent random draws from distribution with zero mean and a known standard deviation. This is a typical multi-comparison setting, that is dressed by considering each element of the difference matrix as a step of the random walk.

When comparing two N×N matrices of Spearman’s rank correlation coefficients, one collected in control condition, one in test condition, our null hypothesis states that there are no differences between the two conditions and, therefore, that the observed correlation coefficient differences are only due to statistical fluctuations (the standard error “SE” of the Spearman’s rank correlation being $0.6325/\sqrt{n-1}$). Another way to state the null hypothesis is: the element-per-element difference divided by $\sqrt{2}*SE$ (the “normalized differences”) of the two correlation coefficient matrices has a null mean value with a standard error equal to 1. Our problem becomes then to test if the N*(N−1)/2 normalized differences (the correlation matrix is symmetrical) is compatible with random draws from a distribution with mean 0 and a standard deviation equal to 1. To answer this question, we draw a parallel to a random walk: each element of the upper diagonal part of the normalized differences matrix is seen as a step of a random walk and the sum of the steps is then the distance from the origin reached by the random walk. We just have to decide once and for all in what order we add these steps and further, we need to construct a domain that will contain a given fraction, say 95%, of the random walks (whose steps are interdependently and identically distributed with a mean 0 and an SD 1, we neither want nor need a Gaussian hypothesis here). If we can build such domain, we will accept the null hypothesis when an observed random walk is entirely contained with the domain and reject it otherwise. Donsker’s theorem guarantees that a properly normalized version of our random walk converges toward a canonical Brownian motion process under the null hypothesis. The work of Kendall et al. (2007) gives the expression of the boundaries of the minimal surface domain containing a given fraction of the observed random walks. They also show that a boundary of the form ± ($a+b*\sqrt{t})$ gives rise to an almost minimal surface domain while being much easier to compute. We used the latter in this work (Kendall et al., 2007; Pouzat et al., 2015). All the details, codes and data related to this test are available at the following address: https://github.com/christophe-pouzat/haeger-et-al-face-processing-in-developmental-prosopagnosia. See also the Supplementary Methods for more details.

#### Step 4: Analysis of Representational Similarity Analysis Matrices Over Time

The Donsker’s test results in a global evaluation of the difference between the two groups in the specific conditions. In a second step, we further aimed at exploring whether participants were able to maintain the level of correlation, evidenced with RSA, over time.

Here, we applied a data driven approach. We observed that control subjects maintained a high level of correlation during the entire paradigm, expressed as high correlations all throughout the matrix (Figure 3, “Results” section), whereas DP failed to do so. We therefore intended to probe whether correlation levels were sustained both during short trial intervals (i.e., trial distance) and long trial interval.

For this aim, we plotted the average values of all rows aligned on the diagonal of the lower triangular matrix, yielding a correlation metric for different trial distances. For example, given *T* correct trials and thus a resulting *T* × *T* correlation matrix, the Δ-th diagonal below the main diagonal contains the values of interest (for the given trial distance Δ = *l* – *k*, with row index *k* and column index *l*). These values correspond to a fixed trial distance interval and allow analyzing the remaining correlation for these different trial distances. With *T* fixed and given Δ, *T - Δ* related correlation matrix entries exist for the diagonal, which were then averaged over median for demonstrating the effect of time and trial sequence on the voxel correlations in right FFA. The procedure can be explained as follows


$$r_{i}=\frac{1}{n-1}\sum_{j=1}^{n-i}\gamma_{n-j+1,j+i}$$

Statistical comparison of single-data points for different trial distances was made via Mann-Whitney-*U*-test and Bonferroni-Holm-correction for sufficient underlying data points of at least six (until *Δ = 10).*

Note that a trial-distance of “10” represents a relative time interval, as only correctly answered trial were considered, and trials of low, medium and high memory conditions were intermixed during the run of the experiment.

#### Step 5: Association With Behavioral Results

In order to associate the results of our RSA with the behavioral results of our experiment, we performed an exploratory analysis in calculating Spearman’s rank correlations between mean trial distances of all the subjects and performance and respective reaction time of all subjects for the high load condition. This was done by half-splitting the trial distances into odd and even *Δ.* This analysis yielded significant results for even Δ, and therefore allowed to perform the same analysis on the odd mean trial distances of all the subjects for encoding and for maintenance of the high load condition.

#### Voxel-Based-Morphometry and Region-of-Interest Structural Analysis

In order to rule out that functional differences in face processing brain areas might be due to marked morphological differences between both groups, we further performed a VBM-analysis. This was done in CAT12 toolbox^3^ (Version r1364) implemented in SPM12. 3D T1-weighted images were normalized, following non-linear registration, and consequently segmented into gray matter, white matter and cerebrospinal fluid components. The segmented, modulated, and normalized gray matter images were then smoothed using an 8-mm full-width-half-maximum Gaussian kernel. Additionally, the total intracranial volume (TIV) was estimated. After, a flexible full factorial analysis was performed, with age, gender, and total intracranial volume as covariates. Gray matter and white differences of contrast controls > prosopagnosic subjects were consequently reported at family-wise error (FWE)-corrected level at *p* < 0.05 as well as uncorrected at *p* < 0.001.

As a further step, gray matter volume of the right fusiform gyrus, derived from the neuromorphometrics segmentation atlas (provided by Neuromorphometrics, Inc.^4^ in CAT12 was estimated and compared between both groups directly via Student’s *t*-test.

#### Data Availability

The detailed description, code and data for the RSA as well as the t-maps from the functional localizer are available in a separate GitHub-depository.^5^.

## Results

### Poorer Memory Performance in Developmental Prosopagnosia

The prosopagnosic group performed worse than the control group irrespective of the task difficulty during the Sternberg paradigm.

A mixed repeated measures ANOVA with the factors condition (low, medium, high load) and group (DPs vs. CTL) revealed for performance a main effect of condition [*F*_(__2, 46)_ = 31.14; *p* = 2.81⋅10^–9^, *d* = 2.33] and group [*F*_(__1, 23)_ = 6.64; *p* = 0.017, *d* = 1.07] and a trend for an interaction [*F*_(__2, 46)_ = 2.44; *p* = 0.098, *d* = 0.65]. Similarly, the same mixed repeated measures ANOVA for reaction time revealed a significant effect for condition [*F*_(__2, 46)_ = 36.82; *p* = 2.83⋅10^–10^, *d* = 2.53] and for group [*F*_(__1, 23)_ = 11.0, *p* = 0.0031, *d* = 1.38], but no interaction [*F*_(__2, 46)_ = 0.27; *p* = 0.77, *d* = 0.21]. An overview of the consequent detailed statistical comparisons between the different memory load condition for performance and reaction time is given in the data Tables 1A,B. Performance and reaction time for control and prosopagnosic participants for the three load conditions (low, medium, high load) are shown in Figure 1B.

**TABLE 1:** Overview of the statistical comparisons via Student’s *t*-test with Bonferroni-Holm-correction for the different memory conditions and groups.

	**Comparison of memory conditions**		*p*-value *	***t*-value**
**(A)**				
Mean performance in % (standard deviation) *All groups*	Low load 92 (10)	Medium load 88 (9)	0.059	2.5
	Low load 92 (10)	High load 77 (11)	**5.39⋅10^–6^**	6.84
	Medium load 88 (9)	High load 77 (11)	**3.25⋅10^–4^**	5.14

Mean performance in % (standard deviation) *Control subjects*	Low load 93 (9)	Medium load 92 (5)	0.64	0.48
	Low load 93 (9)	High load 83 (8)	**1.5⋅10^–3^**	4.18
	Medium load 92 (5)	High load 83 (8)	**1.9⋅10^–3^**	4.07

Mean performance in % (standard deviation) *Prosopagnosic subjects*	Low load 91 (11)	Medium load 84 (10)	**5.6⋅10^–3^**	3.37
	Low load 91 (11)	High load 71 (11)	**6.55⋅10^–5^**	5.97
	Medium load 84 (10)	High load 71 (11)	**4.6⋅10^–3^**	3.47

Mean performance in % (standard deviation) *Controls* vs. *prosopagnosic subjects*	Low load controls 93 (9)	Low load prosop. 91 (11)	0.50	0.69
	Medium load controls 92 (5)	Medium load prosop. 84 (10)	**0.014**	2.67
	High load controls 83 (8)	High load prosop. 71 (11)	**0.0072**	2.95

**(B)**				
Mean reaction time in ms (standard deviation) *All groups*	Low load 1.27⋅10^3^ (412.38)	Medium load 1.52⋅10^3^ (436.41)	**4.81⋅10^–7^**	7.85
	Low load 1.27⋅10^3^ (412.38)	High load 1.58⋅10^3^ (466.87)	**8.75⋅10^–8^**	8.67
	Medium load 1.51⋅10^3^ (436.41)	High load 1.58⋅10^3^ (466.87)	0.40	1.54

Mean reaction time in ms (standard deviation) *Control subjects*	Low load 1.04⋅10^3^ (219.33)	Medium load 1.26⋅10^3^ (259.93)	**7.0⋅10^–3^**	4.42
	Low load 1.04⋅10^3^ (219.33)	High load 1.33⋅10^3^ (242.42)	**1.81⋅10^–5^**	9.14
	Medium load 1.26⋅10^3^ (259.93)	High load 1.33⋅10^3^ (242.42)	0.40	1.62

Mean reaction time in ms (standard deviation) *Prosopagnosic subjects*	Low load 1.48⋅10^3^ (441.32)	Medium load 1.75⋅10^3^ (440.61)	**1.73⋅10^–4^**	6.79
	Low load 1.48⋅10^3^ (441.32)	High load 1.82⋅10^3^ (507.18)	**1.6e⋅10^–3^**	5.27
	Medium load 1.75⋅10^3^ (440.61)	High load 1.82⋅10^3^ (507.18)	0.40	0.88

Mean reaction time in ms (standard deviation) *Controls* vs. *prosopagnosic subjects*	Low load controls 1.04⋅10^3^ (219.33)	Low load prosop. 1.48⋅10^3^ (441.32)	**0.035**	3.12
	Medium load controls 1.26⋅10^3^ (259.93)	Medium load prosop. 1.75⋅10^3^ (440.61)	**0.017**	3.35
	High load controls 1.33⋅10^3^ (242.42)	High load prosop. 1.82⋅10^3^ (507.18)	**0.029**	3.04

*In Table 1A performance for the different memory conditions is illustrated and in Table 1B reaction time in ms. Highlighted in bold font are the significant results.*

*Bonferroni-Holm-corrected.

In sum, participants with DP showed a worse performance during the Sternberg paradigm compared to the control subjects and also had longer reaction times, increasing with difficulty of the task. Mean reaction time but not mean performance over all conditions was further negatively correlated with CFMT performance (*r*_*s*_ = −0.42, *p* = 0.038).

Concerning positioning dependence, prosopagnosic subjects had higher absolute accuracy for recognition of faces that had been shown at the last position (80%) vs. those shown in first position (65%) during the high load condition. In contrast, controls had higher accuracy for first positions (86%) vs. last positioning (79%). However, this finding did not reach significance in a mixed ANOVA (see Supplementary Tables 2A,B).

### Conventional Activation-Based Analysis: Fusiform Face Area-Activation as a Function of Memory Load and Group

**FIGURE 4:**
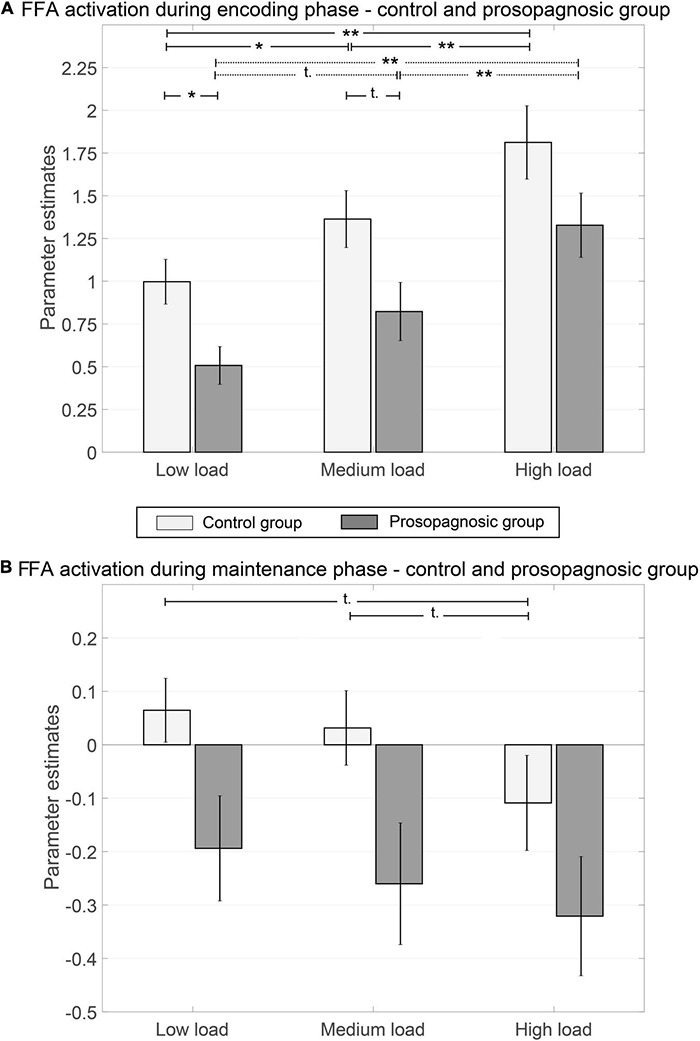
Parameter estimates of right FFA for control and prosopagnosic subjects during encoding **(A)** and maintenance phase **(B)** of the different memory conditions including standard error of the mean; *(*p* < 0.5), **(*p* < 0.01), ***(*p* < 0.001), t, trend; if not otherwise stated not significant.

We used the individual right FFA mask from the functional localizer to further examine group differences between prosopagnosic and control participants during the Sternberg paradigm. A mixed repeated measures ANOVA with factors group (DP vs. CTL) and condition (low load, medium load, high load) on right FFA activation yielded for the encoding phase a significant effect of group [*F*_(__1, 23)_ = 5.82, *p* = 0.024, *d* = 1.01] and a significant effect of condition (low, medium, high memory load) [*F*_(__1.61, 37.09__)_ = 38.02, *p* = 1.80⋅10^–10^, *d* = 2.55]. There was no interaction [*F*_(__1__.61_, _37.09__)_ = 0.055, *p* = 0.95, *d* = 0.10] (see Figure 4A). For the maintenance phase, the same ANOVA showed that these effects persisted, namely with a significant effect of group [*F*_(__1, 23)_ = 4.51; *p* = 0.045; *d* = 0.89] and a significant effect of condition [*F*_(__2, 46__)_ = 4.52; *p* = 0.016; *d* = 0.89] with no interaction [*F*_(__2_, _46__)_ = 0.31; *p* = 0.73; *d* = 0.23] (see Figure 4B).

Global FFA-activation thus increased with memory load, both in CTL and in DP. However, the absolute activation level of the FFA was lower in DPs both during face encoding and maintenance.

To assess whether this response was selective for the FFA region, we probed the activation during the task with a similar procedure as for the FFA masks in another adjacent area of the visual system, the right parahippocampal place area (PPA), which we derived from house stimuli with the functional localizer. As expected, both for the encoding and maintenance phase, the activation in the PPA showed neither group differences [*F*_(__1, 23)_ = 1.36; *p* = 0.26; *d* = 0.49; *F*_(__1, 23)_ = 0.053; *p* = 0.82; *d* = 0.10] nor memory load differences [*F*_(__2, 46)_ = 0.045; *p* = 0.96; *d* = 0.09; *F*_(__2, 46)_ = 1.10; *p* = 0.34; *d* = 0.44].

### Representational Similarity Analysis Within Fusiform Face Area—Global Effects

RSA was used to dissect the neural representations of correctly answered face processing trials within the FFA. For this aim, we computed the correlation of the beta values of each voxel inside the right FFA, separately for memory condition and group.

**FIGURE 5:**
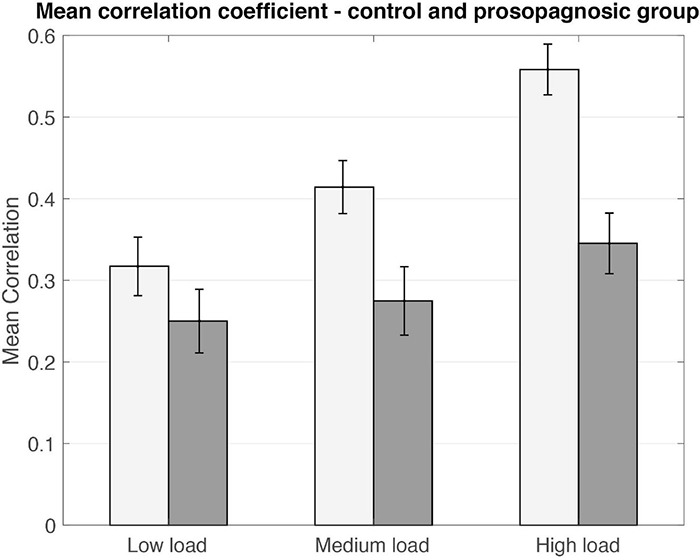
Mean correlation coefficients plotted for control and prosopagnosic group for the different memory conditions including standard error of the mean. Statistical comparison is performed via Donsker’s analysis.

As a first result, the mean inter-trial correlation increased with increasing task difficulty, analogous to the global FFA-activation (Figures 3, 5). In control participants, correct task responses in the high load condition were paralleled by uniformly high inter-trial correlation level. The neural representations evinced with RSA are thus clearly linked to face memory demand (Figure 3).

Correlation matrices for control and DP participants are shown in Figure 3. Inspection of the matrices in Figure 3 suggested two main findings that were probed separately in the following. First, the global correlation level differed between control and DP participants, with diminished inter-trial correlation for the latter. Second, control-participants maintained a high level of correlation all throughout the experiment for the high-load condition, whereas in DP, higher correlations were clustered for neighboring trials and dropped off with trial distance.

### Global Correlation Levels Derived From Representational Similarity Analysis Are Lower in Developmental Prosopagnosia

**FIGURE 6:**
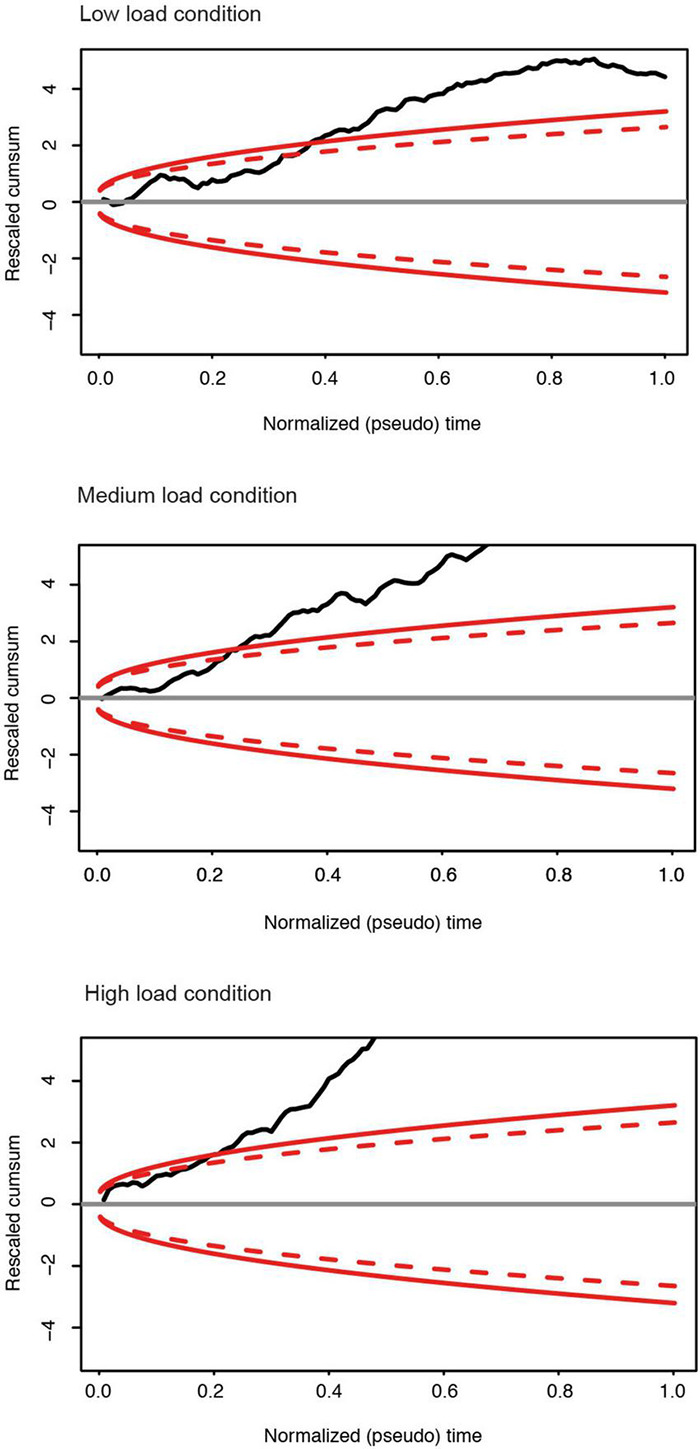
For the different memory load conditions in the maintenance phase: Rescaled cumulative sum of the row portions of the upper triangular part of the differences matrix of each subject group (DPs vs. Controls). Red continuous: boundary of the 0.99 domain; Red dashed: boundary of the 0.95 domain. For all memory conditions, there is a significant group difference of the correlation matrices between the control and prosopagnosic subjects.

To corroborate the finding of unequal correlation levels, we compared individual matrices based on Donsker’s theorem (Figure 6). The null-hypothesis (no difference between the matrices) can be rejected if the random walk algorithm exceeds the 99% interval of a Brownian motion. This analysis allowed to confirm group differences in all memory load conditions at *p* < 0.01. In line with the mean correlation coefficients, we can therefore conclude that DP participants show a lower level of correlations as compared to the control group. Corresponding results were obtained for the encoding phase for all load conditions (see Supplementary Figures 4, 5). To exclude an impact of possible cluster size of the activated FFA, we repeated Donsker’s analysis on the encoding phase for a subsample of 8 subjects from the control and prosopagnosic group with size-matched FFA cluster, yielding similar results.

### Developmental Prosopagnosia Fail to Maintain High Correlation Levels Over Time

Two main findings emerged from the RSA. The first was the increase in correlation level with increased memory load, which was deficient in DP (section “DP Fail to Maintain High Correlation Levels Over Time”). The second was an unexpected finding of different dynamics of correlation over time. While in control subjects, the high level of correlation was sustained over time, i.e., over the entire matrix, DP showed high correlations only in the proximity of the diagonal, i.e., during adjacent trials. We therefore set out to probe this second finding, a decrease in correlation level over time in DP, by deconstructing the correlation metric as a function of trial distance. Within our correlation matrix, we attributed to each trial its distance from the diagonal, i.e., a trial distance of 1, 2, 3 until 10. Note that in absolute time, a trial distance is a relative value (see section “Materials and Methods”).

**FIGURE 7:**
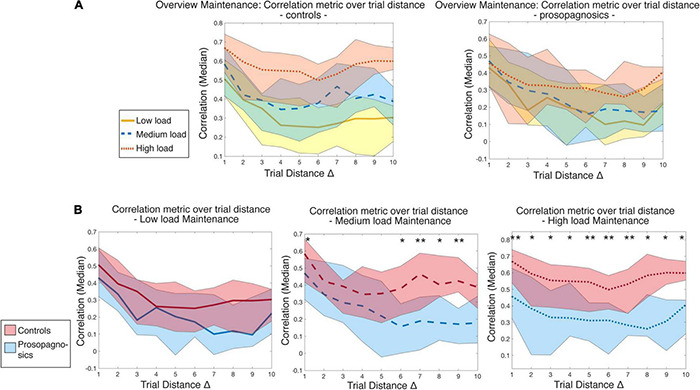
Illustration of correlation metric over trial distance with **(A)** all memory conditions for controls vs. prosopagnosic subjects and **(B)** low, medium, and high load conditions with statistical comparison between controls and prosopagnosics for maintenance phase. Trial distances are statistically compared for each Δ of each memory load condition between control and prosopagnosic subjects until *Δ = 10 via* Mann-Whitney-*U*-test and Bonferroni-Holm-correction *, (*p* < 0.05), ** (*p* < 0.01). The colored areas represent the range between the 10% and 90% percentile of the specific diagonal entry distribution.

When focusing on the maintenance phase of Figure 7, a clear waning of the correlation level with trial distance emerged. The diagonal in our matrix is defined as a trial distance of Δ = 0 (the trial correlated with itself). Correlations were high among neighboring trials and then rapidly decayed with trial distance. This effect was most pronounced for the low load condition in control participants (Figure 7A, yellow line). Indeed, there was less waning for the medium load condition and subjects maintained the highest level of correlation with the highest perceptual demand. The three conditions were indeed separate in control participants, but not so in DP (Figure 7A, right panel). DP participants showed comparably higher correlations of directly adjacent trials, possibly due to shortly maintained neural representation, followed by a marked drop off.

Apart from the low load condition, group differences between CTL and DP were evident in the medium load, especially with increasing distance, and mostly pronounced in the high load condition, where both at short and long trial distances, mean correlation levels were significantly different (corrected for multiple comparisons, Figure 7B). To rule out that this was attributed to possible attention decreases over time, reaction time of the correct trials in the prosopagnosic group was checked over the course of the high load condition and did not increase.

### Correlation Levels Are Linked to Reaction Time and Performance

To establish the link between the RSA and behavioral results, mean correlation coefficients of the odd trial distances of our encoding as well as maintenance phase were associated with the behavioral results of the paradigm. There was a significant negative correlation with reaction times for the most demanding high load condition during maintenance (*r*_*s*_ = −0.47, *p* < 0.001) with a trend for correlation with performance (*r*_*s*_ = 0.17, *p* = 0.061). Significant association between mean correlation of the odd trial distances and reaction time was similar during the encoding phase of the high load condition (*r*_*s*_ = −0.26, *p* = 0.0033), but not for performance (*r_*s*_* = −0.056, *p* = 0.53).

In sum, correlation of functional activity within the FFA, sustained over time, was deficient in DP and explained poorer performance in working memory for faces (Haeger et al., 2018).

### Voxel-Based-Morphometry and Region-of-Interest Volumetric Analysis

VBM analysis did not reveal morphological significant volume differences in gray and white matter between our control and prosopagnosic group both at *p* < 0.05 FWE-corrected as well as uncorrected at a liberal threshold of *p* < 0.001 in the fusiform gyrus. Also when regarding on regional volume level, there was no volumetric difference in right fusiform gyrus, when comparing controls and prosopagnosic right gray matter volume in the right fusiform gyrus [*t*(23) = 0.58, *p* = 0.57, *d* = 0.23].

Given the sample size of our study, this analysis essentially permits to rule out the contribution of macroscopic anatomical differences to our main functional results.

## Discussion

The present study examined the neural underpinnings of face processing in DP via Representational Similarity Analysis (RSA) within the right FFA.

DP-participants evinced poorer performance and increased reaction times in the Sternberg memory paradigm compared to control subjects. In the conventional fMRI analysis, we found that activation of the right FFA during face encoding and maintenance increased with tasks difficulty. The neural signature of the FFA was examined with RSA in order to dissect correlations between individual voxels. Again, the global level of correlation between correctly answered trials increased with higher memory demand. This effect was equivalent in the encoding and the maintenance phase.

The correlation matrices shown in Figure 3 demonstrated two major findings:

(1). The global correlation level was different between controls and DP, as confirmed with a random walk analysis. DP participants demonstrated lower correlation levels within the voxels of the FFA.
(2). There was an effect of time, CTL were capable of sustaining the level of correlation all throughout the experiment. The higher the task difficulty, the more homogeneous the correlation level over time. Conversely, activity in the FFA of DP-participants showed a marked drop-off in correlation with increasing trial distance.

These two major findings held for both the face encoding and maintenance phase. Sustained correlation levels over time were linked to shorter reaction times, and in trend to better task performance.

Altogether, in DPs, neural representations within the FFA are deficient during face encoding and maintenance. Both the global level of correlation and its maintenance over time are diminished, which is suitable to explain poorer performance and reaction times. Interestingly, alterations in the FFA seemed to be solely functional, since we could not find morphological differences between our groups via VBM analysis even at liberal thresholds and in our atlas-based volumetric analysis of the right fusiform gyrus.

### Face-Processing Within the Fusiform Face Area Is Affected in Developmental Prosopagnosia

The present study joins a series of studies corroborating evidence that the FFA is malfunctioning in DP (Dinkelacker et al., 2011; Furl et al., 2011; Zhang et al., 2015). Diminished functional activity of the FFA is quite a homogeneous finding in DP (Williams et al., 2007; von Kriegstein et al., 2008; Dinkelacker et al., 2011; Furl et al., 2011; Németh et al., 2014; Rivolta et al., 2014; Zhang et al., 2015; Witthoft et al., 2016; Jiahui et al., 2018). Furthermore, alterations of the N170, a highly time resolved biomarker of face-selectivity, also argue in favor of atypical neural processing within what is regarded as the core system (Bentin et al., 1999; Kress and Daum, 2003; Towler et al., 2016).

Moreover, it is widely acknowledged that the fusiform and occipital modules of face processing are integrated in complex circuits that are at least partly controlled via top-down control of higher order association complex (Zhu et al., 2011; Eimer et al., 2012; Lee and Geng, 2017; Elbich et al., 2019; Lin et al., 2019). In the light of ample evidence on network curtailing in DP (Thomas et al., 2009; Dinkelacker et al., 2011; Song et al., 2015; Rosenthal et al., 2017), it would therefore be surprising to find a “preserved” FFA function in DP. A recent study on functional connectivity of the FFA supports this notion (Zhao et al., 2018).

Our data underline the necessity to go beyond the global activation level in order to appreciate the complex neural signaling within the FFA.

### Neural Representations Within the Fusiform Face Area Are Less Robust in Developmental Prosopagnosia

Zhang et al. (2015, 2016) have yielded an account of how to glean information from computational analysis. They had used multivariate pattern analysis in the right FFA in order to probe whether DP were sensitive to face-configural information. Our approach scrutinizes two aspects of neural coding, (i) similarity of representations within the FFA and (ii) the dimension of sustained activity over time.

Similarity of representations are expressed in the global correlation level across correctly answered trials, which increased with memory demands, thus corroborating its relevance for the task. Our study did not tap into the nature of the stimulus encoded, but we may hypothesize that the highly correlated signaling within the FFA conveys at least in part the configural characteristics of the face, as described by Zhang et al. (2015). In their study, they were able to decode face configural processing from FFA activation in control subjects, meaning that FFA activation is highly robust and predictable, but not so in DP. We would therefore speculate that the fine-tuned pattern necessary for face processing expresses face expertise. One example of genuinely acquired expertise was given by Gauthier et al. (1999) in their study on “greebles,” i.e., animal heads with facial features. While these stimuli did not activate the FFA in naïve participants, they did so in highly trained “greeble experts”, what Gauthier et al. (1999) attributed to neuronal recruitment.

If we extrapolate these findings to our data, a high degree of similarity of representation may reflect face-identity processing (Axelrod and Yovel, 2015), i.e., the expertise that the FFA acquires for faces or equivalent stimuli during brain maturation (Nordt et al., 2018). And finally, for adult observers, the FFA-activation achieves a surprising stability across repeated scanning sessions (Nord et al., 2017), which may be related to a specific cytoarchitecture of face vs. place-selective areas (Weiner et al., 2017).

In this sense, control subjects in our sample might succeed in configural decoding, as described in Zhang et al. (2015), while the prosopagnosic subjects fail to do so, which is also reflected by poorer behavioral performance.

In sum, a high level of correlation within the FFA is compatible with its highly specialized role in face-processing.

### The Fusiform Face Area-Response to Increasing Memory Load Is Insufficient in Developmental Prosopagnosia

Most of the studies on face-processing in general, and more so in DP, have focused on the FFA-activity at stimulus onset and its relation to the subject’s face-recognition capacity. However, between the perception of a face and its anchorage in the long-term memory lie a multitude of processing steps in time and space. Our study does not intend to fully resolve these steps but to better apprehend the role of stimulus maintenance in the FFA.

Our data convey two aspects of face processing in DP. The global response of the FFA to increasing memory load and the neuronal signature during stimulus maintenance.

The modulation of memory load, either in a *n*-back task or in a Sternberg paradigm, is a standardized protocol for probing face memory in the FFA (Jha and McCarthy, 2000; Druzgal and D’Esposito, 2001, 2003; Axmacher et al., 2007, 2008, 2009; Lepsien et al., 2011), with increased activation correlating with better performance (Lepsien and Nobre, 2007). The effect of load on fMRI activity both in the encoding and maintenance phase could well promote the concept of working memory processing in the FFA. Interestingly, these fMRI studies have recently been confirmed by a transcranial-magnetic-stimulation study showing that stimulation of the right FFA increased face working memory performance (Brunyé et al., 2017).

Our study demonstrates that DP are impaired in face processing related activation, consistent with their poorer behavioral performance. While control participants had strong increases in FFA activation with increasing task difficulty, DP did not attain the same level both in face perceptional encoding and in the maintenance phase. Note that our data convey the notion of deactivation during the maintenance phase. This is not an uncommon finding in fMRI studies on the subject, which might concern various brain regions (Schon et al., 2009; Stretton et al., 2012; Németh et al., 2014). Deactivation in the fusiform gyrus during maintenance can be explained by diminished functional connectivity with the hippocampus and inferior frontal gyrus (Rissman et al., 2008; Lohse et al., 2016; Albonico and Barton, 2019), another possible explanation might be different BOLD dynamics in DP (Németh et al., 2014). Yet, the apparent deactivation might also reflect methodological approaches in baseline determination within the General Linear Model.

Our data on diminished FFA response in DP indicate that face memory is deficient in the various time scales of face perception and processing (our current study), short-term (CFMT), and long-term memory (diagnostic interview).

Obviously, our paradigm did not tap into the contribution of individual perceptional differences to the current results. As a matter of fact, interindividual variability in perception may be genetically determined (Etzel et al., 2020).

### Maintenance of Representations and Memory Performance

Our first main finding, the higher the memory demand, the higher the global correlation level, is well in line with the long-standing notion of the FFA in working memory for faces (Druzgal and D’Esposito, 2001, 2003; Brunyé et al., 2017). As a second main finding, our data underline the importance of maintenance of representations.

In terms of short-lived stimulus maintenance in the order of 1,500 ms, there is a gradient of posterior to anterior electrocortical signal decay in the visual system (Gerber et al., 2017). Our data showed that maintaining a high degree of similarity within the FFA over the order of minutes related to memory performance and distinguished control participants from DP. Indeed, working memory for faces has been shown to be impaired in DP (Jackson et al., 2017).

We would suggest that the deficiencies in the perceptual maintenance of similarity within the FFA are suitable to explain poorer memory in DP. Probably, they are not exclusively generated in the FFA but express ongoing network interaction of top-down and bottom up signaling (see above).

### Interactions of Perception, Working Memory, Long-Term Memory, and Mental Imagery

Maintenance of a stimulus representation is crucial for memory performance, and will finally facilitate long-term memory (Axmacher et al., 2007; Haeger et al., 2015). Our data hints to a behavioral-neuronal relationship during face processing within a single trial, reflected in maintenance deficits over several trials linked to performance and CFMT-scores (which correspond to face learning in the realm of minutes) leading consequently to probable anamnestic long-term face recognition impairment being typical for DP. We would suggest that deficient similarity coding within the FFA is a key element in the sequence possibly leading to long-term face memory curtailing.

As a final thought, we would like to extrapolate the concept of deficient maintenance to mental imagery (Ishai, 2010). People with DP have the lowest self-reported mental imagery published so far (Grüter et al., 2009), analogous to acquired prosopagnosia following damage to the right FFA (Barton and Cherkasova, 2003). DPs often dream imageless and cannot conjure up precise facial representations, as opposed to object imagery (Tree and Wilkie, 2010). Mental imagery not only activates the FFA, but is sufficiently stable to be decoded via computational pattern analysis (Kriegeskorte et al., 2007; Reddy et al., 2010).

As an outlook on the intriguing dimensions of face-processing, we suggest that deficient maintenance of FFA-signaling over time is a common denominator for impairments in perception, working and long-term memory as well as mental imagery.

### Limitations of the Study

As for the majority of studies on DP, the sample size of our study might limit the statistical power of our analysis, notably in the relationship of RSA and behavioral performance, with remains on the level of a statistical trend. VBM analysis was performed both on the whole brain and in a region of interest analysis of the right fusiform gyrus from our atlas-based volumetric analyses, in contrast to previous studies (Garrido et al., 2009; Dinkelacker et al., 2011). Yet, given the sample size, it might still be underpowered to unravel subtle differences in cortical volume. Further studies on larger cohorts will be needed to examine the interaction between functional and possibly structural differences in DP.

We are aware that other groups consider the CFMT as single diagnostic test, while we and others (Zhang et al., 2016) rely on the diagnostic interview. Hence, while DP scored less than control participants in CFMT, the two diagnostic approaches are not strictly superposable. Our current data demonstrate that DP are deficient in various levels of face memory, from perception and working memory (Sternberg paradigm), to short-term (CFMT), and long-term memory (anamnestic interview). Analogous to other neurological diseases, we consider it appropriate to base a clinical diagnosis on a group of characteristic features and tests, rather than on one single test.

And finally, for the theoretical and methodological reasons mentioned in the introduction, we limited the RSA analysis to the right FFA, pinpointed notably as a major component of face identity processing. Obviously, these data integrate into a complex network of face processing (Duchaine and Yovel, 2015), and should on the long run be confronted with similar analyses of the ensemble of face sensitive brain areas.

## Conclusion

Our study on signaling in the right FFA demonstrates that the DP lack robustness in the neuronal signature of face processing. Participants with DP failed to obtain high correlation levels within the FFA and to maintain these representations over time, in line with their behavioral impairment. We suggest that highly stable similarity coding within the FFA is an expression of face expertise. Deficient maintenance of high-level encoding relates to poorer memory performance and may ultimately explain curtailing in long-term memory and mental imagery in DP.

## Data Availability Statement

The datasets presented in this study can be found in online repositories. The names of the repository/repositories and accession number(s) can be found below: https://github.com/christophe-pouzat/haeger-et-al-face-processing-in-developmental-prosopagnosia.

## Ethics Statement

The studies involving human participants were reviewed and approved by the University of Muenster, Germany (Protocol No. 3XKenn2) and University of Bonn, Germany (Protocol No. DI 1217/2-1). The patients/participants provided their written informed consent to participate in this study.

## Author Contributions

VD, NA, IK, CE, and AH conceived and designed the study. AH, VD, CP, VL, and KN’D analyzed the data. VD, NA, and AH interpreted the data. AH drafted the manuscript. VD, NA, IK, CP, KN’D, VL, and CE edited and reviewed the manuscript. All authors contributed to the article and approved the submitted version.

## Conflict of Interest

The authors declare that the research was conducted in the absence of any commercial or financial relationships that could be construed as a potential conflict of interest.

## Publisher’s Note

All claims expressed in this article are solely those of the authors and do not necessarily represent those of their affiliated organizations, or those of the publisher, the editors and the reviewers. Any product that may be evaluated in this article, or claim that may be made by its manufacturer, is not guaranteed or endorsed by the publisher.

## Footnotes

1
https://www.nitrc.org/projects/wfu_pickatlas/

2
http://www.fil.ion.ucl.ac.uk/spm

3
http://www.neuro.uni-jena.de/cat/

4
http://Neuromorphometrics.com/

5
https://github.com/christophe-pouzat/haeger-et-al-face-processing-in-developmental-prosopagnosia

## Abbreviations

ACPC: Anterior commissure-posterior commissure
BOLD: Blood-oxygenation-level-dependent
CTL: Control subjects
DP: Developmental prosopagnosia
EPI: Echo-planar-imaging
ERP: event-related brain potential
FFA: Fusiform face area
fMRI: Functional magnetic resonance imaging
FWE: Family-wise error
FWHM: Full width at half maximum
MNI: Montreal Neurological Institute
ROI: Region of interest
RSA: representational similarity analysis
SD: Standard deviation
SEM: Standard error of the mean
SPM: Statistical parametric mapping
TE: Echo time
TI: Inversion time
TR: Repetition time
WM: Working Memory
WMA: World medical association.
