# Supplementary material for: Face Processing in Developmental Prosopagnosia: Altered Neural Representations in the Fusiform Face Area
Source: Front Behav Neurosci. 2021 Nov 18;15:744466. doi: 10.3389/fnbeh.2021.744466 (PMC8636799; doi:10.3389/fnbeh.2021.744466)
Supplement: Supplementary file 1 [file Data_Sheet_1.pdf]

Supplementary material

1. Supplementary methods

1.1 Diagnosis of Prosopagnosia

1.1.1 Detailed questionnaire for Developmental Prosopagnosia (DP):

Overview of the 21-items questionnaire for assessing prosopagnosia, with items rated on a five-point Likert scale.

Face recognition
- 01 I can easily follow actors in a movie
- 02 I often do not recognize people who I know
- 04 People often tell me I do not recognize them
- 05 I can decide immediately if a face is familiar
- 07 I always recognize family members
- 12 I recognize famous people immediately
- 13 I sometimes recognize people I do not know
- 19 I avoid meetings as I might overlook familiar people
- 20 I do not recognize people the day after a brief meeting

Learning/memorizing individual faces
- 06 It takes me a long time to recognize people
- 20 I do not recognize people the day after a brief meeting

False positive and false negative rates of face recognition
- 13 I sometimes recognize people I do not know
- 04 People often tell me I do not recognize them

General facial information, such as gender, physical attractiveness, and emotions
- 14 I can decide immediately whether a face is male or female
- 17 I can see if a face is attractive
- 18 I have problems to read emotions in a face

Demonstrating the presence/absence of inner images of familiar faces and/or objects
- 11 I can easily form pictures of close friends in my mind
- 10 I can easily form a mental picture of a red rose
- 08 I can easily find things, which are not in the right place
- 15 I can easily walk backwards in a room

Complex pattern recognition on the example of orientation
- 16 I get lost in new places

Using strategies
- 09 I mostly recognize people by their voices

19 I avoid meetings as I might overlook familiar people

Socialization

21 I have much contact with other people

Heritability

03 Some of my family have problems in recognizing faces

1.1.2. Main aspects of the diagnostic interview

Main aspects of the diagnostic interview for DP (Grüter et al., 2007; Johnen et al., 2014; Kennerknecht et al., 2008; Stollhoff et al., 2010, 2011) (see also Methods section in main manuscript):

(1) Uncertainty in face recognition: The leading symptom, which affected people report, is an irritating lack of confidence in the recognition of faces and the inability to learn new faces to an extent that they can easily be recognized. Other symptoms include not recognizing familiar people unexpectedly or in crowded places, confusing unknown persons with familiar persons. Only anecdotal mentioning of not recognizing people was not taken as a positive criterion.

(2) Significantly prolonged recognition time for faces.

(3) Development of compensatory strategies as sign of a longstanding and frequent problem: (i) adaptive behaviour to cope with face recognition impairment; Persons with prosopagnosia rely heavily upon other personal characteristics, like voice, gait, clothing, etc.. (ii) Avoidance behaviour: avoidance of places other people could be met unexpectedly, being first to an appointment in a restaurant, or looking absent minded whenever walking in the street; (iii) Giving explanations such as: “Sorry I have forgotten my glasses” or “I was absent minded”.

(4) Repeated anecdotal stories of events such as having overlooked familiar people were found to be extremely helpful. Persons with prosopagnosia also typically have problems in following the actors in a movie especially when the scenes change frequently or the characters are similar (e.g., mixing up different inspectors in detective/crime films).

1.2 FMRI-Analysis

1.2.1 Design matrix of the functional localizer

Figure S1

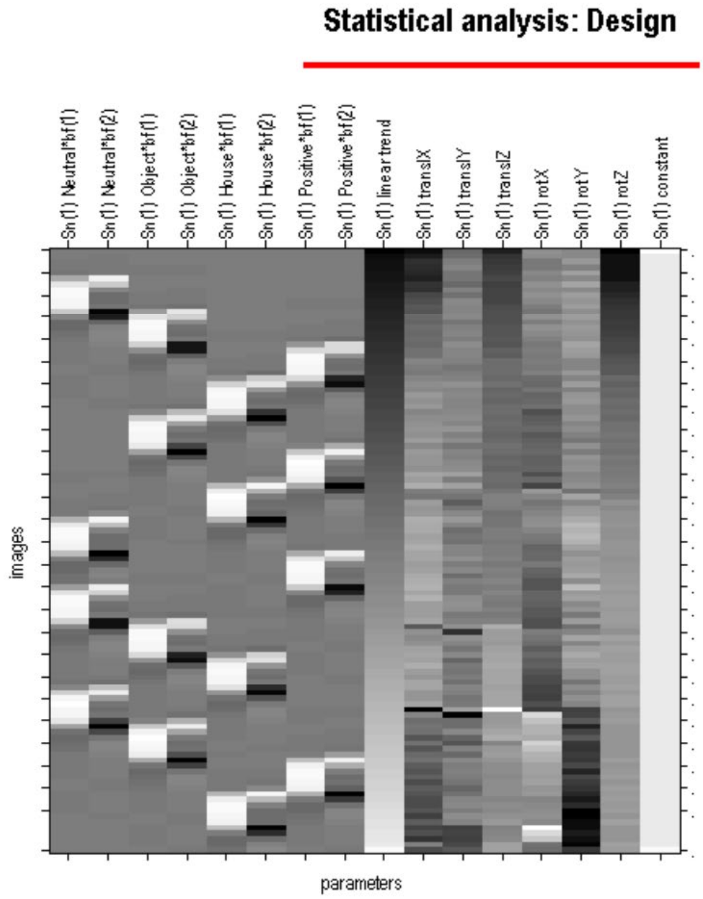

Figure S1) Design Matrix of the functional localizer in block design (neutral faces, objects, houses and positive faces), including time derivatives for each main regressor as well as additional movement regressors, the linear trend and the constant.

1.2.2 Peak coordinates of the right Fusiform Face Area (FFA) for all subjects

Tables

S1

**Group**	Subjects	t-value	Peak Coordinates x y z
Controls	C1	3.23	40 -36 -24
	C2	5.99	38 -42 -20
	C3	5.24	42 -48 -24
	C4	2.94	30 -42 -20
	C5	7.14	36 -46 -12
	C6	5.48	34 -48 -18
	C7	13.13	48 -52 -16

	C8	7.55	40 -50 -12
	C9	10.63	38 -54 -12
	C10	11.90	40 -54 -18
	C11	12.91	42 -54 -16
	C12	4.51	34 -34 -22
Prosopagnosics	P1	3.60	42 -46 -22
	P2	8.18	36 -42 -24
	P3	4.06	36 -70 -20
	P4	3.07	34 -44 -18
	P5	8.87	42 -42 -16
	P6	6.66	36 -42 -16
	P7	6.67	42 -62 -18
	P8	6.52	44 -50 -20
	P9	4.16	36 -66 -12
	P10	4.99	38 -44 -20
	P11	9.52	38 -44 -18
	P12	4.93	40 -60 -16
	P13	3.05	52 -42 -18

Table S1: Overview of the peak coordinates of the right FFA for each subject, derived from the face localizer, results of contrast all faces versus objects and houses.

1.2.3 Design matrix for the conventional analysis of the Sternberg-Paradigm

Figure S2

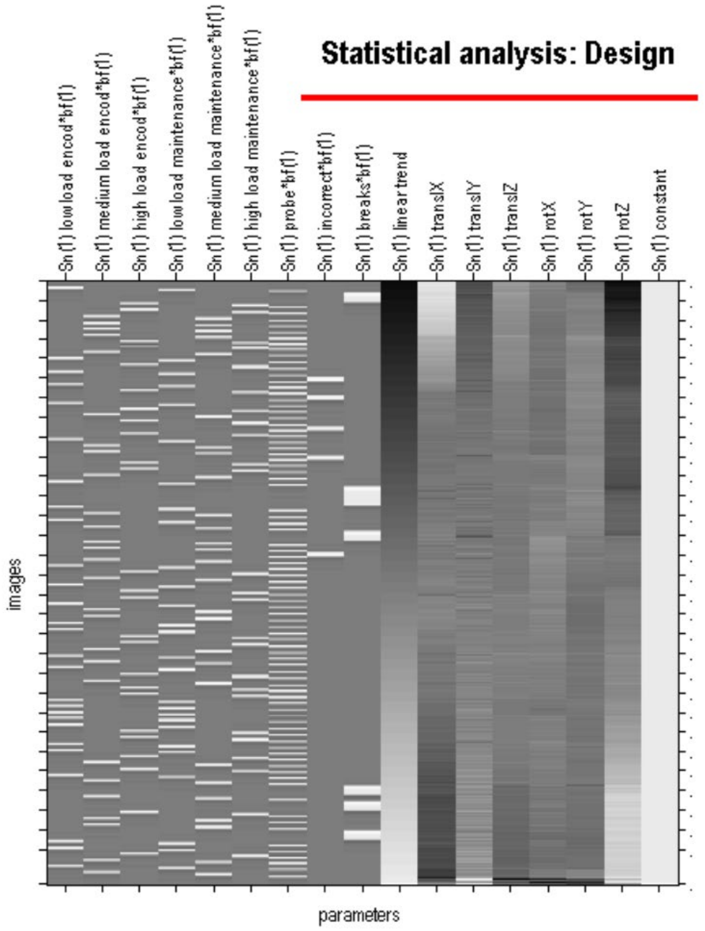

Figure S2) Design Matrix of the main experiment, modelling as regressors the encoding phase (regressors 1-3) for low, medium and high memory load, as well as the maintenance phase for all three conditions (regressors 4-6), modelling further the probe as 7th regressor as well as all incorrect trials and the breaks. Further regressors include movement parameters, the linear trend and the constant.

1.2.4 Design matrix for the Representational Similarity Analysis (RSA) of the Sternberg-Paradigm

Figure S3)

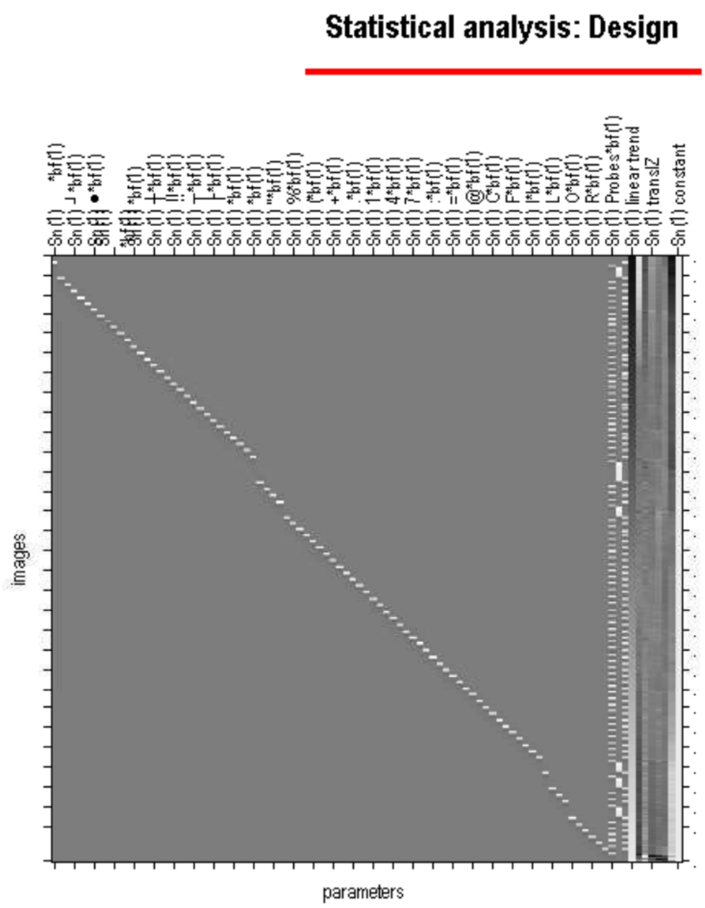

Figure S3) Design Matrix of Representational Similarity Analysis including all trials as regressors with their maintenance time. One trial includes all encoding phases, one all probes, one all breaks and the movement parameters as covariates.

2. Supplementary Results

2.1 Behavioral Results

2.1.1 ANOVA of the behavioral performance during the Sternberg-Paradigm depending on image position

S2A

Source	SSq	df	MSq	*F*-value	*p*-value
Between-subjects factor	0.065	1	0.065	0.75	0.39
Between-subjects error	2.00	23	0.087	-	-
Within-subjects factor	0.008	1	0.008	0.14	0.72
Within x between interaction	0.13	1	0.13	2.12	0.16
Within-subjects error	1.42	23	0.062	-	-

S2B

Source	SSq	df	MSq	*F*-value	*p*-value
Between-subjects factor	96928.10	1	96928.10	0.301	0.59
Between-subjects error	7416114.68	23	322439.77	-	-
Within-subjects factor	1756090.17	1	1756090.17	3.764	0.065
Within x between interaction	2276.28	1	2276.28	0.005	0.94
Within-subjects error	10731944.96	23	466606.30	-	-

Table S2A and B:

Results from the mixed ANOVA for factors group (controls vs. propopagnosic subjects) as between-subjects factor and the within-subjects factor position (1st position vs. 4th position). S2A for task performance, S2B for reaction time; SSq: Sum of squares, df: degrees of freedom, MSq: mean squares, F-values, p-values.

2.2 Imaging Results

2.2.1 Comparison of RSA group matrices via Donsker analysis

Detailed Explanation of the Donsker analysis:

If the null hypothesis (no differences in matrices between groups) is correct, the normalized differences matrix should follow a Gaussian distribution, more precisely a t-distribution, with a null mean value and a variance equal to 1: $\frac{\hat{\gamma}_{i,j}^{CTL}-\hat{\gamma}_{i,j}^{PRO}}{\sqrt{\hat{\sigma}_{i,j}^{CTL}/n^{CTL}+\hat{\sigma}_{i,j}^{PRO}/n^{PRO}}}$. For each matrix element (i,j), we use the previously computed mean $\hat{\gamma}_{i,j}^{CTL}$ and variance $\hat{\sigma}_{i,j}^{CTL}$ value (computed on n^{CTL}subjects) for the control group and a mean $\hat{\gamma}_{i,j}^{PRO}$ and a variance $\hat{\sigma}_{i,j}^{PRO}$ (computed on n^{PAT} subjects) for the group of prosopagnosics. The degrees of freedom of this t-distribution are given by

$$\nu \approx \frac{\left(\hat{\sigma}_{i,j}^{CTL}/n^{CTL} + \hat{\sigma}_{i,j}^{PRO}/n^{PRO}\right)^2}{\hat{\sigma}^{4}{}_{i,j}^{CTL}/(n^{2CTL}\,(n^{CTL}) - 1) + \hat{\sigma}^{4}{}_{i,j}^{PRO}/(n^{2PRO}\,(n^{PRO}) - 1)}.$$

The null hypothesis corresponds to the assumption that the upper triangular part of the differences matrix can be generated by drawing independently for each element a random number distribution with a mean value of 0 and a variance of 1. We are therefore facing a multiple-comparison problem.

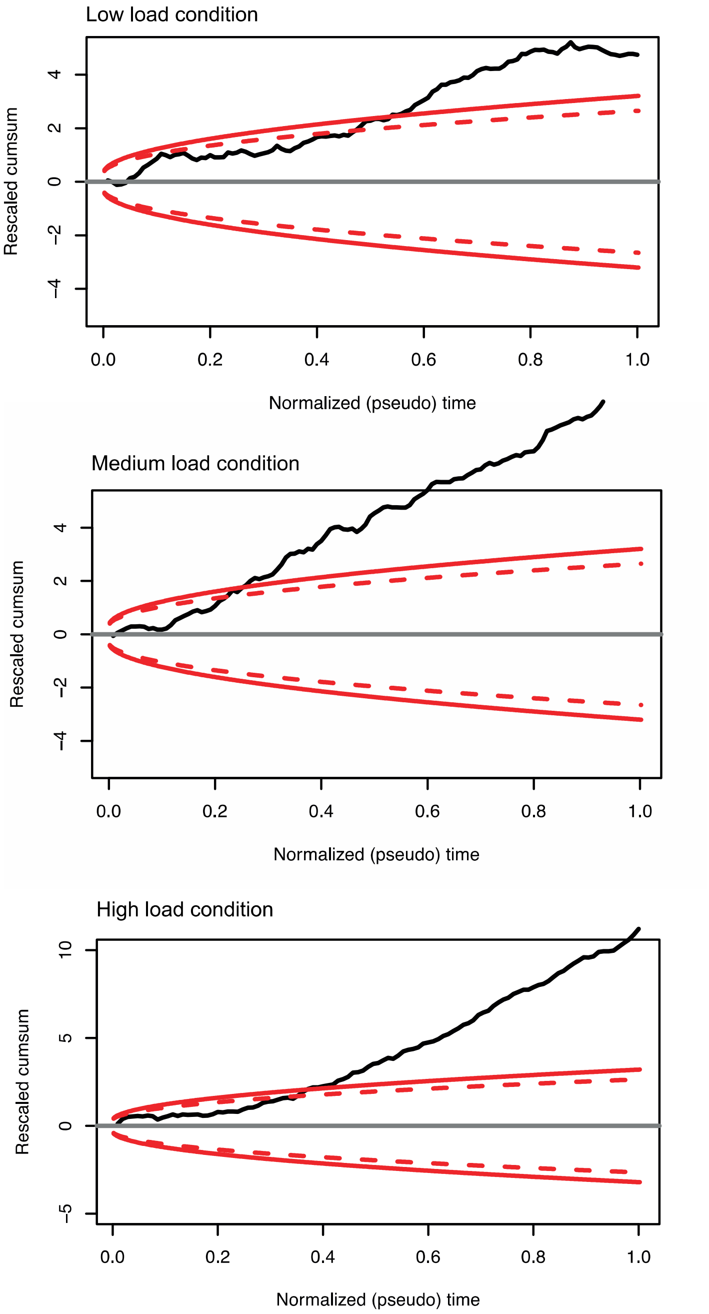

Fig. S4: For the different memory conditions encoding phase: Rescaled cumulative sum of the row portions of the upper triangular part of the differences matrix of each subject group. Red continuous: boundary of the 0.99 domain; Red dashed: boundary of the 0.95 domain. For all conditions, there is a significant difference between the control and prosopagnosic group.

2.2.2 RSA trial distance analysis: Maintenance of neural representations

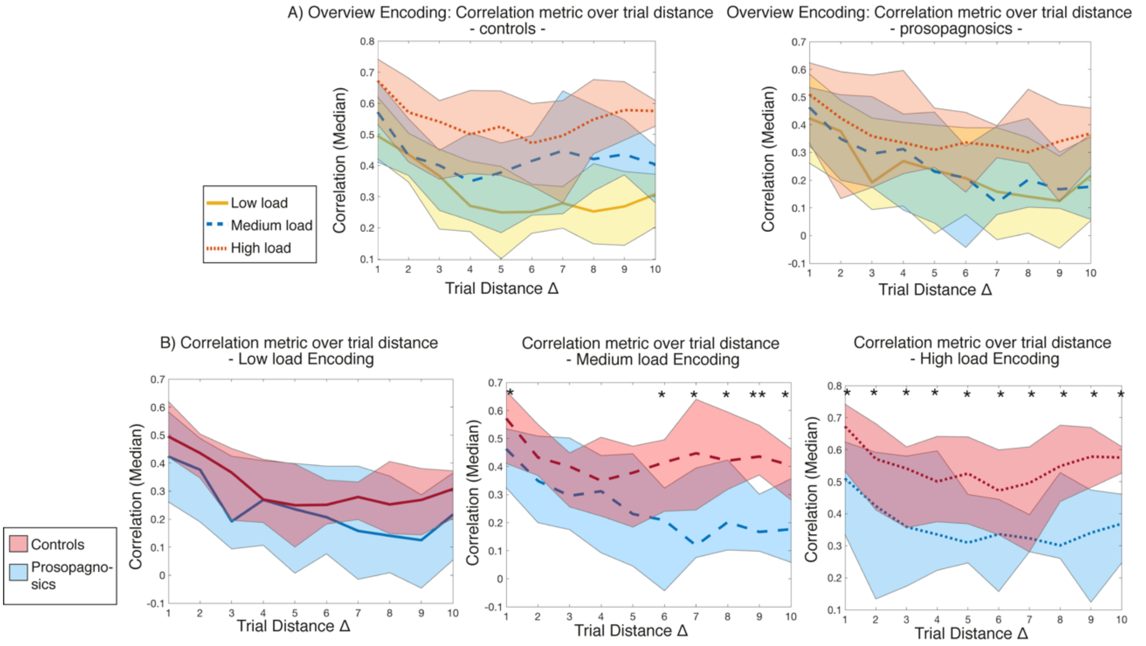

Fig. S5: Illustration of correlation metric over trial distance with (A) all memory conditions for controls versus prosopagnosic subjects and (B) low, medium and high load conditions with statistical comparison between controls and prosopagnosics for encoding phase. Trial distances are statistically compared for each *Δ* of each memory load condition between control and prosopagnosic subjects until *Δ = 10 via* Mann-Whitney-U-test and Bonferroni-Holm-correction (* ($p < 0.05$), ** ($p < 0.01$)). The colored areas represent the range between the 10% and 90% percentile of the specific diagonal entry distribution.
